# What does coercion in intensive care mean for patients and their relatives? A thematic qualitative study

**DOI:** 10.1186/s12910-022-00748-1

**Published:** 2022-02-05

**Authors:** Susanne Jöbges, Corine Mouton Dorey, Rouven Porz, Bara Ricou, Nicola Biller-Andorno

**Affiliations:** 1 Institute of Biomedical Ethics and History of Medicine, University of Zurich, Winterthurerstrasse 30, 8006 Zurich, Switzerland; 2 University Hospital Bern, 3010 FreiburgstrasseBern, Switzerland; 3 Department of Anaesthesiology, Clinical Pharmacology, Intensive Care, and Emergency Medicine, University Hospital of Geneva, 1211 Geneva, Switzerland

**Keywords:** Autonomy, Coercion, Formal–informal, Intensive care unit, Ethics, Patient experiences

## Abstract

**Background:**

The need for an ethical debate about the use of coercion in intensive care units (ICU) may not be as obvious as in other areas of medicine, such as psychiatry. Coercive measures are often necessary to treat critically ill patients in the ICU. It is nevertheless important to keep these measures to a minimum in order to respect the dignity of patients and the cohesion of the clinical team. A deeper understanding of what patients and their relatives perceive during their ICU stay will shed different light on intensive care management. Patients' experiences of loss of control, dependency and abandonment may lead to a new approach towards a broader approach to the concept of coercion in intensive care. The aim of our research is to explore the experiences of patients and relatives in the ICU and to determine when it might be possible to reduce feelings and memories of coercion.

**Methods:**

We conducted and analysed 29 semi-structured interviews with patients and relatives who had been in the ICU a few months previously. Following a coding and categorisation process in MAXQDA™, a rigorous qualitative methodology was used to identify themes relevant to our research.

**Results:**

Five main themes emerged: memory issues; interviewees’ experiences of restricting measures and coercive treatment; patients’ negative perception of situational and relational dependency with the risk of informal coercion; patients’ perceptions of good care in a context of perceived dependency; progression from perception of coercion and dependency to respect for the person. All patients were grateful to have survived. However, coercion in the form of restraint, restriction of movement, and coercive treatment in the ICU was also acknowledged by patients and relatives. These included elements of informal coercion beyond restraints, such as a perceived negative sense of dependence, surrender, and asymmetrical interaction between the patient and health providers.

**Conclusions:**

To capture the full range of patients' experiences of coercion, it is necessary to expand the concept of coercion to include less obvious forms of informal coercion that may occur in dependency situations. This will help identify solutions to avoid or reduce negative recollections that may persist long after discharge and negatively affect the patients' quality of life.

**Supplementary Information:**

The online version contains supplementary material available at 10.1186/s12910-022-00748-1.

## Background

The need for an ethical debate on the use of coercion in intensive care may not be as obvious as in other fields of medicine, such as psychiatry. However, important questions arise regarding not only the use of coercive measures in the ICU, but also the scope and meaning of coercion [1].

Coercion can be defined “as a mode of influence that operates by threats and force; aims at controlling the recipient’s being, movement, or will; and leaves, at least initially, its recipient disadvantaged” [2]. This includes overcoming a person's free will or expression of the will of an incompetent person, sometimes called "natural will" [3]. Incompetency is a frequent phenomenon in the ICU—patients often lack decision-making capacity so that proxies are in charge of decision-making esp. When there is no advanced care planning (ACP) or advanced directive [4].

Agitation, delirium, confusion, disorientation and drowsiness are common in the ICU and obtaining an informed consent concept is often not applicable [5]. The justification for the use of restraining measures in the ICU is to protect the patient from self-harm by removing the devices [6–8]. Intensive care treatment includes monitoring, life support and medical therapies, with the goal of saving the life of a critically ill patient. For the patient, it means forsaking freedom of action and being dependent on life-sustaining equipment. Thus, the life-threatening situation of the seriously ill patient may lead to a perception of powerlessness, loss of control, loss of freedom and loss of self, and consequently to a sense of dehumanisation and disembodiment [9]. Depending on the culture in the ICU and the nurses’ experience and training, there is wide variation in when and how physical restraints are used in the ICU [8, 10–14]. In this context, standards have been developed to guide the use of restraining measures [15–17]. A better understanding of patients’ experiences could contribute to the development of guidelines and training concepts to reduce the use of coercion in the critical care setting. However, there is a lack of literature about patient experiences of restraining measures in the context of intensive care treatment [1]. The concept of coercion is an important subject of study in psychiatry, which goes beyond a framework of what is legal or not [18] with an estimated prevalence of 29–59% [19]. Informal coercion includes situation of persuasion, undue influence on the patient, inducement and threat [18]. Patients in psychiatry describe the temporary situation of illness and incapacity and perceived formal coercion but also describe informal coercion as deception, withholding information, threat, and the absence of communication, cheating, ignoring the patient and various situations to manipulate the patient [19–21]. There is a gradation of potential coercive measures that reflects the situation in intensive care units, where patients' serious illness is accompanied by a labile state of dependence and incapacity due to, for example, coma, sedation, disorientation. From the patient's perspective, the perception of pressure measurements such as influence, manipulation and threat is often subjective due to their incapacity in the ICU. Clinical teams tend to underestimate the possibility of the occurrence of the informal coercion in acute medicine where attention is concentrated on formal coercion [22]. A focus on restraining measures may be too narrow to fully capture patients’ experience of coercion in the ICU. Coercion can come in different forms and variations, which need to be clarified as the literature often has confusing wording. In our qualitative research we used the following classification:

Formal coercion includes liberty restricting measures and coercive/compulsory treatment [23]:

- Restricting measures such as physical restraint, chemical restraint and psychological restraint [24]. Likewise, restrictions due to the environment (bedspaces, lights, noise, absence of cognitive simulation) can be perceived as restraints by the patient [25].
- Coercive treatment, a treatment against the *expressed or shown (natural) will* used to maintain or restore health under coercion, especially when there is a risk of self-harm or harm to others [16, 23]. Coercive treatment in the ICU can be observed, for example, in the areas of anti-infective therapy, transfusions or early mobilisation.

Informal coercion:

- Situations, such as dependency, lack of privacy that patients are subjected to and consequently perceive as being forced upon them [2, 3].
- Patients’ feelings of deception, influencing through withholding information, absence of communication, cheating, or being ignored as well as being threatened [19, 20].
- Patients’ subjective reports of any adverse or traumatic experiences are also classified under informal coercion.

**Fig. 1:**
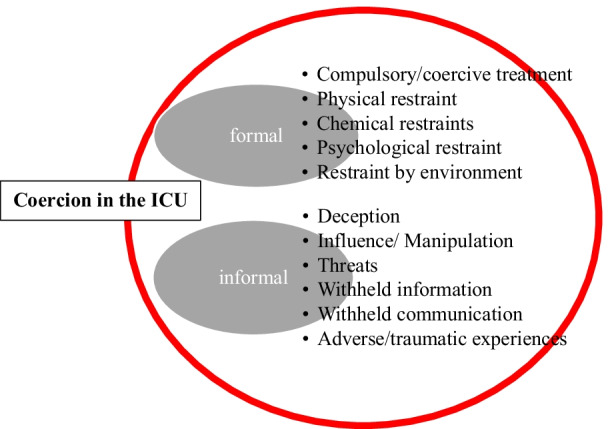
Working concept of coercion in the ICU

Figure 1 summarizes these different possibilities of coercion.

The use of coercion in a clinical setting requires not only legal compliance, but also medical and moral justification, including a weighing up of benefits and risk reduction against induced harm [23, 26]. Patients describe the situation of dependency in the ICU with feelings such as powerlessness, frailty, and vulnerability [27, 28]. These rather negative associations might be explained, at least in part, by the high social value of autonomy in our Western societies [29].

As a factor affecting individual autonomy, coercion does not only include restrictions of freedom through restraining measures [16, 23, 30]. Conceived more broadly, informal coercion can be defined as restricting the freedom to express oneself, to define and pursue one's goals, or to have options for action [30]. This would include measures such as “the application of overt or covert force by psychological means, either directly, in contact with a patient, or indirectly, with the involvement of relatives or other relevant persons.” [16].

In the particular context of intensive care treatment, a mismatch has been identified between the competence and power of the team and the patient's perception of helplessness and dependency (“powerlessness and counter-power” [30]). Motivated by the team’s desire to apply the best therapy for the patient, care in the ICU may involve, consciously or unconsciously, the use of informal coercion. Therefore, critically ill patients in an intensive care unit are at risk of being exposed to and perceiving coercion. It would therefore be important to be able to assess this risk at the patient level to better judge the proportionate benefit of care. A possible difficulty in using patients' experiences of formal and informal coercion stems from the fact that their recollections of their time in an intensive care unit are subjective, often perceived as somewhere between reality and delusion [13, 31]. Still, these recollections are part of patients’ memories of this particular episode in their life. They will affect how patients judge the care they received and how well they are able to cope with the experience without a lasting negative impact on their quality of life. A deeper understanding of patient experiences, such as loss of control, loss of autonomy, helplessness and dependency has the potential to refine our thinking about the concept of formal and informal coercion, and ultimately our management of coercive measures in the ICU.

Our paper addresses the lack of data on patient experiences in the ICU. Following standards of good qualitative research (SRQR) [32], we present a qualitative study with semi-structured interviews of former ICU patients and relatives. The aim of our study was to probe patients’ and relatives’ perspectives on different forms of coercion perceived during the ICU stay to help inform best practices with a view to use of coercion in intensive care.

## Method

In order to provide a better understanding of experiences with physical restraints and other coercive measures by ICU patients, a thematic analysis, derived from Grounded Theory, was chosen to explore the individual experience and reflections of people who stayed in an ICU [33].

The study successfully passed the internal review board of the Institute of Biomedical Ethics and History of Medicine (CEBES 19072017) and received a waiver from the competent ethics committee to be conducted nationwide Canton of Zurich, Cantonal Ethics Committee (KEK Req. 2018-00050) and was conducted in accordance with the Declaration of Helsinki [34].

The methodology follows the standards set by the Health Experiences Research Group (HERG) at the University of Oxford [35]. Semi-structured interviews were conducted, analysed and included in the Swiss chapter of the International Database of Patients’ Experiences research initiative (DIPEx International), a digital platform allowing results to be shared with patients facing a similar experience, as well as health professionals and policymakers, and following a rigorous academic standard [35, 36].

### Sampling and recruitment of participants

To include a wide diversity of experiences, a maximum variation purposeful sampling was used for the selection of participants in the three main Swiss language regions. Sample size was determined by data saturation, i.e. the point at which additional data fails to generate new information. A series of 30 interviews was foreseen. Recruitment was carried out with the support of hospitals and rehabilitation centres in the cantons of Aargau, Basel, Bern, Geneva, Schwyz, St. Gallen, Vaud, Wallis and Zurich, through the first author’s direct contacts and by snowballing Patients were informed about the study and invited to participate by the intensive care physician during their routine 6-month follow-up visit after discharge. All patients who agreed to participate and to give their contact information to be contacted by our research team, were then given detailed information about the study, and were given the free choice to participate or not, and to freely withdraw at any time. Study information sheets and informed consent forms were produced in 4 languages: French, English, Italian and German, and sent to all potential participants meeting the inclusion criteria, i.e.

- patients with somatic diseases, or close relatives who were involved in patient care,
- over 18 years of age,
- patients who had spent more than 72 h in an intensive care unit in the previous 6–12 months.

The study excluded patients who were unable to participate in an interview because they had significant residual brain damage or lacked the capacity to consent to participating in the study.

### Interview process

At the time of interview, the information sheet—which had been made available before the interview appointment—was explained to the interviewee, who could then ask supplementary questions before filling in and signing a first informed consent form that permitted recording (audio with or without video) of the interview and use of anonymized data for research.

The interview topic guide (supplement: interview guideline) was developed with the help of a scoping review identifying an initial framework about the concept of coercion in ICU (Jöbges et al. submitted reference). The literature review showed the importance of patients' feelings of dependency in intensive care, so the more structured part of the interview sought to better reveal the patients' feelings. The topic guide included three parts (see Additional file 1):

1. Participants’ personal experience of ICU. Assessed with open-ended questions.
2. More specific questions about recollections of restraints, bad dreams, hallucinations, negative or positive feelings regarding dependency, environment and shared decision-making. After the first few interviews, the topic of communication emerged as crucial for a better understanding of patients’ situations and was added to the initial interview guide.
3. Possible recommendations for other patients, relatives and clinical teams.

The topic guide was reviewed by external experts in intensive care and qualitative research, as well as native speakers of Swiss languages. To ensure a trusting atmosphere, a methodology of face-to-face interviews, at the interviewees’ location, was chosen. Interview proceedings were recorded in a qualitative research journal of field notes. Five interviews were performed remotely during the COVID lockdown period with a secure system approved by the university data protection board.

Interviews took place between October 2019 and September 2020 and were conducted in German, French, Italian or English, depending on the participant's native language. Most of the interviews were conducted by two of the authors (SJ, CMD). Two other interviewers contributed to Italian and German interviews (see Acknowledgements).

### Data management and analysis

All audio recordings were transcribed verbatim on a continuous basis, and data were exported into MAXQDA™ software for Mac. The recorded and transcribed interviews were handled confidentially. All material was stored in password-protected computer files. Data and documents were saved for secure storage, backed up in the University data archive.

Facilitated by MAXQDA™, coding and categorisation processes were gradually updated. The first 6 transcripts were used for developing the coding book. Codes were added when necessary, with later interviews, and previous interviews were reviewed accordingly to ensure consistent coding for all transcriptions. Memos were written throughout the research process. The first level coding was grouped into categories. The thematic analysis procedure used successive matrices to cross-tabulate different categories of response. Our interpretation followed a mix of deductive (initial framework-informed) and inductive (theory-generating) approaches, with a continuous comparison method to interpret expected and emergent themes [33]. Themes were developed in written English. The analysis followed the OSOP method resulting in a map of key themes presented in the results [37].

### Interpretation standpoint

Two authors (SJ, CMD) coded their respective interview transcripts independently, shared their coding, discussed the different entries, and found a consensus for the coding book; they developed themes and proposed their analysis to the advisory board that was already involved in the scoping review and the study design. One author has a medical background in ICU and further education in bioethics. The second author has a background in medicine, ethics, and qualitative research. Other interviewers (see Acknowledgements) have backgrounds in philosophy, anthropology and nursing. The advisory board members and co-authors have backgrounds in intensive care, biomedical ethics and philosophy.

## Results

### Demographics

The study included 29 participants. Qualitative saturation was recognized after the first 19 interviews; however, the study continued in order to ensure maximum variation. The interviews were conducted with 19 patients who had suffered from medical, surgical or neurological conditions, 3 patients with a Covid infection requiring mechanical ventilation, and 7 relatives. Table 1 shows the demographic data.

**Table 1:** Demographic data

*Age*	
20–30	1
30–40	5
40–50	3
50–60	7
60–70	5
70–80	7
> 80	1
*Sex*	
Female	13
Male	16
Patients	22
Relatives	7
Language	French: 10, German: 18, Italian: 1
Diseases	Medical Surgical neurological
*Length of interview*	
13–99 min	37 min

### Findings

The interview data are rich and go beyond the scope of a single publication. The focus in this paper is on categories that could evoke a notion of coercion or risk of coercion as expressed by the patients themselves or alternatively their relatives. The perspective is that of the patients or their relatives and not that of the health professionals. The thematic analysis identified five themes that highlight the perception of patients and relatives regarding the different possible forms of coercion in the ICUs and introduce possible improvements in the practice.

1. Memory issues/gaps
2. Interviewees’ experiences of restricting measures and coercive treatment
3. Patients’ negative perception of situational and relational dependency with the risk of informal coercion
4. Patients’ perceptions of good care in a context of perceived dependency
5. Progression from perception of coercion and dependency to respect for the person

1. Memory issues/gaps

Most of the patients were in a comatose state, a few because of their illness, but most often they were put into an artificial coma or deep sedation within the care process in the ICU. One of our interviewees did not even know she was in the hospital. She remembers becoming aware that she did not know where she was:

[…] where am I? I couldn't understand it at all, where they then told me I was in the hospital. (CMI 16).

The absence of memories is itself a source of insecurity for patients who gradually discover part of their history through their entourage. This was also the case for another interviewee, who was only able to make connections six months after his hospitalisation as to what had happened during his stay at that time:

And then [six months later, assessment appointment] I discovered things that I didn't remember or maybe I wasn't aware of at the time. I was surprised to learn that. (CMI 01).

There is an intertwining of subjectivity and objectivity in the reported experiences. As a result, what patients remembered about their stay in the ICU is incomplete, or for some of them mixed up with dreams, hallucinations and delusions. One patient speaks about the difficulty distinguishing between reality and hallucinations:

I don't know if it was my imagination or if they really tied my arms… So I don't know if it's my imagination or if it was/it really happened. (CMI 17).

When they remember dreams, a restlessness, it is overall a “not so good experience". One of our interviewees clearly says that it was not "easy", quite the opposite:

I was just dreaming…And for me it was always a restlessness and just not necessarily such good experiences… Not easy - yeah, it's just… for me it's been a bit of a pain. (CMI 19).

Patients do, however, report certain experiences, which although possibly related to hallucinations, give rise to a memory of feelings that were real, even several months after hospitalisation. One patient was remembering changes in taste and smell; from today's perspective, he can attribute this to the morphine and tell retrospectively:

And I was on morphine quite heavily. And that gave me all sorts of problems, uhm one, the most severe, was the sense of smell and of taste.

This also led to quite unpleasant experiences, such as nausea and vomiting:

And each time I would take a glass of even (water) down, orange juice, I would be vomiting, because of the strength of the taste (CMI 03).

In summary, we can state that all our interview partners have/had to struggle with memory gaps. This means that reproduction of the details and contexts of their stay in the ICU is anything but trivial for them. Their stories are a mix of what they seem to have experienced themselves, what was added afterwards by relatives or doctors, or feelings/emotions to which they give words retrospectively. Regardless of these limitations, it is important to remember that these memories represent real experiences for the patient.

2. Interviewees’ experiences of restricting measures and coercive treatment

We grouped under this theme all patient perceptions of restriction of their freedom of movement (also affecting the freedom to rest and sleep).

Physical restraints are closely related to the loss of freedom of movement because the patient really is tied up, or because medical devices prevent movement.

If you can't move anymore, then it's similar to being tied down. (CMI 08).

One patient uses the metaphor of a prison:

They tied my hands…It's like being in prison. (CMI 09).

One of our interviewees mentions this inability to move and clearly makes the link with the ties and the discomfort of tracheal intubation:

I was strapped down, so I literally couldn't move at all…Because you got tubes and stuff sticking everywhere and/and the tube in my throat was REALLY annoying. (CMI 22).

Another explains how the gloves were such a nuisance that he still felt it in his dreams.

Yes, the gloves were hell. I have long struggled with the fucking gloves in the dreams. They must have haunted me for three dreams. (CMI 20).

Some patients do remember physical restraints in context with their agitation. For instance, this patient was able to understand the situation:

So, in the end they had to tie me up a little bit so they could (-) do their job. (CMI 23)

Relatives feel the vision of their attached relative in an unpleasant way, even if they can understand it.

With white stuff there around the wrist attached to the bed actually. So that she can't pull the tubes out simply… it was like for the crazy people that we have to tie up… I thought to myself but my God, but what a horror she must be suffering so much. (CMI 04, relative)

One relative explains the need for gloves:

Then he panicked so much that he extubated himself…After that, he also got gloves like that. (CMI 20 relative). Another one thought that these measures of physical restraint could be hidden from the family.

That is also a point that I would have liked to know, I was not told … I overheard that by chance. From a nurse who said: Yes, we put gloves on him at night. (CMI 22 relative).

Chemical restraints when mentioned are usually associated with a bad memory. It is difficult to establish coercive treatment from the patient's perspective alone, but it cannot be ruled out.

You're so stuffed with drugs…I didn't like that at all. (CMI 10)

Some patients make the connection afterwards with the medication they received.

I guess due to the fact that they used barbiturate to slow down your brain. (CMI 22),

the morphine, that was also very uncomfortable. (CMI 09).

One of the relatives also comments these measures.

Haldol®, which pretty much sedated him…He only had it at night. (CMI 21 relative)

Combined physical and chemical measures are quite usual, with chemical restraints permitting better acceptance or lightening of physical restraints. Some patients can make the link:

I fought against the gloves so often. But I just couldn't get them off. And um, yeah. Then I always felt the tingling in my head and then I fell asleep. (CMI 20).

Some relatives are aware of this link, too. A daughter was musing about the use of sedative medication despite her mother's refusal:

She [her mother] said she couldn't stand sleeping pills…So why did they have to give it? …because they had to tie her up at some point, I think. (CMI 04 relative)

Psychological restraints cover a very broad spectrum of perceptions. Among them is the feeling of "total pain", which one patient expresses unambiguously:

It's very painful everywhere. And not only the pains of the body, in my psychic mentality, it's not good. (CMI 02).

Another perception is that of being "overruled" or “guilty”:

He [nurse] told me “I have the order to do it, so I'm doing it”. He didn't give me any explanation why he was doing it. …Well, my anger, it even made me feel guilty afterwards, since I apologised. (CMI 05).

Environmental restraints are linked to the machines and the medicalised environment that is unfamiliar and stressful. Most patients describe devices as an obvious cause of restriction.

Machines that with me, all tubes here, the thing under the nose and cables here. (CMI 02);

One is connected to machine… Helpless. (CMI 08)

One interviewee explains how seeing all the tubes can be stressful:

There are tubes everywhere and yes that was … stressful. (CMI 14)

Another one experiences being linked to machines and oxygen as a shock:

In a few days we're like that … That was the shock. (CMI 17)

There are also ICU environmental issues, such as noises and lights, that patients must tolerate with no choice. As it is possible to act on these aspects by improving the environment (e.g. ICU architecture, layout, team behaviours [24]), we have classified as environmental restrictions those which are perceived as restraints when patients complain about them, as in this patient's example:

the noise in the whole building / so in the whole room… That was quite massive. And that had disturbed me … it was very unpleasant. (CMI 14)

Some patients were really bothered by voices coming from other rooms in the ICU, and from other patients:

But there was one patient who was in such a state that he was screaming. And that was painful. So of course, you are full of compassion for the person in question, but at the same time there are moments when you say to yourself but if he could just shut up… On the one hand, compassion and on the other hand, but if he would just shut up! (CMI 01)

Coercive treatment, a treatment against the *expressed or shown (natural) will*, is used to maintain or restore health under coercion, especially when there is a risk of self-harm or danger to others [16, 23]. In the absence of a medical file, we can only refer to it with caution, for instance through the administration of medication, transfusions against the (natural) will of the patient, as in the examples above of a patient refusing blood transfusion (CMI05) and of a relative not understanding why sleeping pills were given to her Mum who had said she could not stand them. (CMI 04).

3. Patients’ negative perception of situational and relational dependency—with the risk of informal coercion

When asked about moments of helplessness, one patient (CMI 22) answers:

Every minute I was there [in the ICU].

Another (CMI 26) concludes:

a feeling of loss of autonomy.

This state of dependence can be experienced negatively by patients through feelings such as helplessness, shame, not being involved in the decision or being misunderstood. Many patients complain about the difficulty of having to bear situational and relational dependency, which can lead to a feeling of loneliness or, on the contrary, aggressiveness:

On one night, I just could not get anyone to come… That was a time when I felt alone, there was nothing I could do. (CMI 03);

I then made myself understood in writing by nodding my head and shaking and striking. Then FINALLY they understood. (CMI 09).

A patient expresses his distress during the whole stay:

It was difficult to find out what they did to you…I got almost zero information about you know why there are 15 tubes stuck into my neck and my arm and everywhere else. Why / what had happened to me during the two weeks that I've been dreaming. Why/why was that happening? (CMI 22)

Such negative narratives in a situation of dependency deserve special attention in that they may be signs of a risk of informal coercion. One of the patients uses the metaphor of war by saying that he had to surrender.

Before, you stood on your own two feet. You did everything on your own. It’s brutal to be so dependent… The worst thing is that you can't talk. You can't communicate… And then you just surrender. (CMI 10)

Even small measures, such as eye drops, can increase the perception of coercion in this situation:

the fact that they would put drops in my eyes and a little bit of cream occasionally, I couldn't see very well. I had/and I really // other than the fact that I (couldn't) see, I was strapped into a bed, it was difficult to um see where I was, … (CMI 22)

Most patients suffer from being medically dependent and powerless because they lose control over the decision-making process:

I was intubated … The woman told me that I wanted to rip the stuff out and so on … All I know is that she was very rude. And that I couldn't articulate myself either. (CMI 18).

Most patients report a sense of frustration with the feeling of dependency:

I was very frustrated because I was getting used to taking these drugs and my psychiatrist [his regular doctor] told me not to stop … so I felt a little powerless. (CMI 06).

It is about feeling powerless, helpless:

I was helpless because I simply could not speak. I could not express myself… Terribly annoying. Terribly effortful in the sense of, yes, it takes effort too. (CMI 11);

for me the worst thing was to be so helpless. It breaks you. (CMI 20).

For some patients the perception of dependency and loss of control is associated with a feeling of fear:

The fear … That was the worst thing for me…But just the fear that someone will hurt me. (CMI 09);

Aggression comes from fear…You are not taken seriously by these people. (CMI 08).

A few patients feel ashamed:

a shameful feeling… I should regain my independence as quickly as possible (CMI13).

Others have a sense of dehumanisation and overruling:

So, what shocked me a bit is, in the ICU, they… when the patient is awake and he's reasonably there, they don't take him seriously. The one doctor, well… this, I really have to say, this is patient-UNWORTHY. (CMI 19)

I am simply no longer perceived as a person. (CMI 24)

Finally, some patients associate dependency and frustration with aspects such as thirst, physiotherapy, noise, sleeplessness: all elements that make them think they are not being taken into consideration.

I almost died of thirst. (CMI 18);

And they'd SPRAY some water in your mouth, but not give you a real drink of water, which only irritated me more. (CMI 22);

They always ignored me, the nurses. They made jokes on the side, and I was lying there so helpless. (CMI 16)

Communication is a major theme in our interviews. Withholding communication or not tailoring information to the patient's level of awareness and understanding can result in a form of informal coercion. Examples are discussed in theme 5, which links this perception of informal coercion to respect for the individual.

4. Patients’ perceptions of good care in a context of perceived dependency

Most patients appreciate the benefit of the care and acknowledge being treated with respect. Some patients report positive or even pleasant memories in an attitude of gratitude to the team:

well, they do it very well, it was admirable, and they really did everything they could to avoid making me feel indebted or whatever. (CMI 01);

The nursing staff, etc., motivated persons who fought, who did everything to make us feel comfortable and especially helped us to get out of the situation. (CMI 26).

These good memories reveal a trusting relationship. One patient uses the word confidence:

I had very many interventions that …I could co-decide …but I have to say I am absolutely/I have confidence in our medical system. (CMI13).

Other patients perceive positively the relational dependency with the medical team in an attitude of acceptance of the situation:

Once you get into the feeling, into the knowledge that you are fairly immobilised, you accept it. (CMI 03);

Since it was to promote my own healing …I applied the instructions. I was a good student. (CMI 07).

One patient expresses the link between accepting the situation and understanding it:

But the nurses took great care of me…I was sweating so much…. They said it was normal because the body must process it and flush out all the medication. …That's how they explained it to me. I don't know if that's true. It sounded logical to me. (CMI 20)

In the context of perceived situational and relational dependency as considered in themes 3 and 4, it should be noted that the patients’ negative or positive perceptions are not exclusive. For instance, patients can remember in the same interview that they felt helplessness and shame and that they received good care. The interviewees often remembered those circumstances in detail, which may either worsen (if perceived as difficult) or alleviate (if perceived as good) their experience of ICU and especially a situation of coercion.

5. Progression from perception of coercion and dependency to respect for the person

Beyond the perception of liberty-restricting measures and feelings of dependency, patients and relatives share experiences directly related to a sense of respect for them. We report interviewees’ insights of what is respect for a person from their perspective. The interpretation is sometimes complex. For instance, statements may express a feeling of gratitude, while at the same time implying what should have been done and yet was not. The data include important hints at details in the care and surroundings of ICU that may be relevant regarding a possible feeling of coercion which can persist as reminiscence, i.e. an account of a memorable experience that can change a person's sense of identity even weeks or months after hospitalisation.

Many patients have very positive memories of the team with the respect to their situation and their person:

Infinite gratitude for the surgeon and the doctors who took care of me at that time, not to mention all the nursing staff, nurses. I would go so far as to say the cleaning staff who are always very pleasant. (CMI 01).

They express gratitude for the care they received in their critical situation and emphasise the professionalism of the team:

Gratitude… and I experienced a lot of beautiful humans, interpersonal beauty. (CMI 13);

And I've never had such good care…I felt I was in good hands. (CMI 18).

Most patients appreciate being reassured, a feeling that they link to a clinical team acting with empathy:

There is a lot of empathy…I think if you need something you can disturb them without it creating a problem. (CMI 17);

you don’t find this sort of team spirit […] outside. (CMI 03).

One of the patients appreciates feeling the positive team spirit:

And these nurses, when they were in the corridors outside the rooms, we could hear them sometimes, if you'll pardon the expression, messing around, eh? They were laughing and all that, and I told them that, that for the patient it does a lot of good. (CMI 23).

Some patients find it useful to write down their experience, to obtain psychological support or to make conscious efforts to think positively because they have survived.

Some kind of debriefing that might have been helpful…maybe a psychologist or a psychologist. (CMI 17);

Just see the positive. You're still alive. (CMI 08).

Other interviewees express their need for an improved communication and wishes for more humane care of a “medical case”, i.e. respect for the person. Communication problems are widely reported by the caregivers themselves. In our patients' interviews, it is possible to capture a demand that is more than communication, that is, attention. Some patients need more attention from the team, which means better interaction, communication or gentleness. A few patients feel neglected or not taken into consideration as a person. Each patient appears to be a person with different needs to care for.

One of the interviewees advises the healthcare professionals on how to approach a patient:

They should approach people slowly… And speak a little slower. And not too loud. Because that scares the hell out of you… And if touch, then please touch on the hands, so that one can also see it. And say, I'm touching you or I'm touching you on the hand now…Don't just come and touch somebody. That scares you and then you switch to defence. (CMI 09).

Another patient regrets that there is not more discussion with the health care team due to lack of time:

But sometimes you should have a little more…to talk, just a little time, but they probably have no time. (CMI 15).

The quality of the presence is also important as it helps most patients to feel secure.

The nurse holding my hand and saying that I was fine… and that calmed me down a lot, to feel that presence at my side. (CMI 23).

Some patients report, in contrast, a presence that ignores them:

they were discussing, these two girls [nurses], without talking to me… I thought it was weird, I said what am I doing here. (CMI 05).

This means that the clinical team should act as if patients were aware of everything, whatever their level of sedation or wakefulness. More generally, what reassures patients is to feel part of the whole process:

That was not discussed with me at all. I think that very little information is passed on here in general anyway. Especially medical information… I would prefer absolute transparency and openness. (CMI 11)

In the interviews with relatives, the same ambivalence is found in the assessment of how attentive and respectful the clinical team was. Some relatives found that the team was very responsive to them, which also can help the patient (CMI 12). Other relatives say that they suffered from a lack of understanding and good communication at the beginning of the hospitalisation in the ICU. One relative regrets not having been included in the care process:

I would have expected a bit more from the hospital. That you as a relative would be supported in such situations… I really felt neglected as a family member…I really would have hoped for more help there…is there someone in the hospital who is there for these situations and would accompany the relatives? (CMI 21 relative).

One relative would like to have more time at first to understand what was going on:

Basically, you arrive at the ICU and walk into the room with no warning. You've never been up there before. You don't know how he's lying there. You don't know how many tubes he has. You don't know what he even looks like. You don't know what he can do, what he can't do. You walk into the room, there is a nurse who says: Can you see him like this? You say: Yes. You don't have any other choice. What other choice do you have? And that's the end of it. No one asked me afterwards. Nobody asked me in the meantime. I didn't even think about psychological care because I didn't have time. (CMI 20 relative).

## Discussion

Our qualitative research provides some original views of the perception of coercion in an ICU setting through the description of patients` and relatives` experiences. Despite their variation in memories, patients describe restraining measures, such as physical, chemical, environmental, psychological restraints, and coercive treatment. In addition to these rather obvious forms of formal coercion, patients describe situational and relational dependencies that were accompanied by positive and negative memories. Dependency and the feeling of not being able to escape the situation were often associated with the perception of disadvantage and informal coercion. The ambivalence of the patients (dependence versus gratitude) in their relationship with the team was regularly addressed. Seeing and respecting the patient as an individual person was described as overcoming these perceptions and was associated with perception of good care by the patients. The patients’ experience of being respected by the clinical team includes being reassured, feeling a presence without it being an aggressive presence, perceiving a good team and trustworthy atmosphere, benefiting from good communication that includes the relatives without any arrogance, and allowing the patient to feel included in the decision-making process. From the patients' perspective, the concept of coercion should be broadened to focus on informal coercion and include the impact of dependency at the situational and relational level.

The health professionals’ perspective on formal coercion has been widely explored in the literature. It is important, therefore, to compare published health professionals’ experiences with the patients’ experiences as illustrated in our study in order to identify convergence and divergence. This might help to define areas for future improvement, especially regarding a framework of situational and relational dependencies which, in an ICU context, would complement the notion of informal coercion in a narrower sense.

### Memory issues

Remembering the time in the ICU is often characterised by confusion, coma or sedation. Delirium, for example, is found in up to 80% of patients in an ICU and subsequently has a negative impact on the course of the disease [7]. Patients often lack mental capacity and it is challenging to establish informed consent [5]. Despite the lack of capacity in our study, patients often remembered formal and informal coercive treatment, with their perception of dependency including good and/or bad experiences. These memories were still present six months after treatment in the ICU.

### Restraining measures -formal coercion

There is a lack of in-depth, qualitative studies on how patients and relatives perceive restraints (Jöbges submitted). To our knowledge, this is the first qualitative interview study exploring patients’ and relatives’ perception of restraints and other forms of coercion in the ICU.

In general, study participants’ memories of perceived coercion were negatively connotated and frightening. The scarce literature available on physical restraints narrates perception of coercion as being very stressful or bothering [25, 38]. Good communication, however, could help patients better accept these physical restraints [39].

Patients’ narratives about non-physical forms of restraint are only described in the context of psychiatry [30, 40]. Looking at commonly used sedation and concepts to treat delirium, there is an overlap with coercive treatment to avoid self-harming by the patients. This form of coercion was addressed by patients in our study. Chemical, but also environmental and psychological restraints were usually mentioned as being associated with bad memories.

The use of coercive measures and coercive treatment is subject to the respective legal requirements and always requires a thorough benefit-risk analysis. Even in the absence of insight into the illness and the need for treatment, coercive measures should always be used as a last resort, after all other possible means have been exhausted [23, 41].

Whether restraining measures can prevent the removal of tubes or other devices is questionable [6, 42]. There are additional risks particularly for physical restraints in the ICU, like an increased need for opioids or benzodiazepines to sedate the patient, which entails a higher risk of delirium and increased mortality [7, 28]. The impact of restraining measures on the development of post-traumatic stress disorder is still under discussion [43, 44].

### Patients’ negative perception of situational and relational dependency—with the risk of informal coercion

The relationship between patients and the healthcare team is characterised by conflicting interests and imbalances. The knowledge and decision-making powers are on the side of the healthcare professionals. Patients perceive a situational or relational dependency, but cannot escape it. For this reason alone, there is a great risk of feeling coerced or disadvantaged in this dependent situation (Jöbges, submitted) [2], often in combination with a sense of ambivalence around the relationship [29].

Patient statements regarding this particular situation need to be interpreted carefully. The situation of being critically ill with memory issues and delirium can influence the perception of coercion. Interestingly, in our study patients who were not sedated during their stay in the ICU, as well as relatives, also had the perception of dependency and dehumanisation. Some patients remembered moments or events in the ICU that are not easily classified either as coercion or as absence of coercion. These negative narratives that related the experience of dependency might best be captured as entailing at least a risk of informal coercion. Negative feelings such as fear, helplessness or shame when not being involved in decisions taken or lack of communication were still present after six months. The interviewees’ major topics were information, communication and respectful treatment. Informal coercion includes a range of practices like situation of persuasion, influencing the patient, inducement and threat [18, 19, 22] that rely on communication within the therapeutic relationship [21]. For some patients, the perception of vulnerability, dependency and loss of control is associated with a sense of dehumanisation, which can be understood as a loss of dignity, autonomy, and self-value [9]. In a situation of powerlessness and dependence, there is a risk that the patient will perceive informal coercion even during routine care if it is not accompanied by special attention and communication. “Coercion occurs only in cases when the receiving party believes they have no choice but to comply” [45]. Examining patients’ experiences identifies situations that might be correlated to adverse influencing and manipulation through withholding of information, isolation and lack of communication, that patients perceive as coercion. Not being able/allowed to define and pursue one's own (therapy) goals or to choose options for action leads to a disadvantage for patients. In some situations when the patient refuses care or treatment in the ICU, inducement can occur when sedation is used, or there is withholding of assistance, or the patient is simply ignored. For instance, to get the patient to accept for his/her own beneficence measures like mobilization or respiratory treatment can be perceived by the patient as coercion. Indeed, our study showed that some patients surrendered their autonomy, or later excused themselves for having opposed the clinical treatment.

The patient’s perception of coercion in the ICU is not only linked to the perception of restraining measures. The situation of dependency leads to a subjective perception of coercion that can be labelled as informal. Including "informal" elements i.e. negatively felt by the patient because of emotional dependency or perceived threat avoidance behavior has the advantage of taking into account a wide range of patients’ experiences and the possibility of uncovering unintentional coercion by the clinical team [19].

### Patients’ perception of good care and progression from perception of coercion and dependency to respect for the person

Most patients also reported positive or pleasant memories and were overall ready to accept what had happened. They appreciated the benefit of the care they had received and acknowledged being treated with respect and having had a trusting relationship. The perception of a good relationship between the healthcare team and the patient made it easier to accept the situation of dependency. Patients mention the feeling of gratitude because they survived and the desire to give something positive back (“repay”) [29]. The expression of gratitude can also mean that the patients feel “themselves as bothering to the healthcare personnel with a lot of needs, and they may be afraid to be seen as overly demanding or ungrateful” [46].

Our study shows how important some details in the care and the ICU environment may be regarding perceptions of formal and informal coercion which can persist as reminiscence, i.e. an account of a memorable experience that can change a person's sense of identity even weeks or months after hospitalisation. Beyond the recollection of liberty-restricting measures, coercive treatment and feelings about dependency, patients and relatives also report experiences directly related to a sense of respect for them. The loss of autonomy and the loss of dignity were always associated with negative feelings.

In the ICU, a clear asymmetry exists between the healthcare team, on the one hand, and the patient and his or her relatives, on the other. However, the clinical team can also suffer when a patient's condition does not improve despite the decisions made in his or her best interest. Balancing the principles of beneficence and non-maleficence may lead to a sense of helplessness [27, 47]. Added to the stressful working conditions in the ICU, such moments can lead to moral distress and burnout [48]. As a recent qualitative study has shown, healthcare professionals, as well as the patients and relatives, strive for humane medicine [27]. There is a discomfort shared by caregivers and patients around the notion of coercion in the ICU [47]. This sentiment is not limited to physical restraints. Apart from the measures themselves, a sense of coercion can arise because of the patient's fluctuating condition and state of consciousness, leading to disorientation and a vague sense of being coerced in this state. The management of patients in such a condition is challenging because it requires a constant adaptation of measures and communication to the clinical situation and to the demands of each patient. Weighing the benefits and risks when using formal and informal coercion, respect for the autonomy and dignity of the patient are the main pillars of humane caring. Respecting dignity, however, should also be understood as including the dignity of caregivers [49, 50].

Caring for patients in the ICU setting is not just about medical outcomes, there are other elements that influence the well-being of critically ill patients and the memories of their ICU stay. Appreciative communication and shared information can clarify and provide a sense of control [51]. This requires time and effort on the part of the medical team, as well as an ethical framework guiding interactions with patients and their relatives [42, 50].

Understanding and respecting patient autonomy in the ICU means “regarding the patients as being capable and having a will of their own” [52]. Patients and relatives could be invited to participate as far as possible in decision-making [52, 53].

In summary, patients wish to be perceived with their narrative individuality; they want to be acknowledged in their current situation of critical illness and to be involved as partners [51]. Respecting the narrative identity of patients, as well as their autonomy, provides a basis for minimising coercion in the ICU [54, 55].

For the patient and the relatives, but also for the healthcare team, intensive care medicine is an extraordinary situation which may include elements of informal coercion beyond liberty-restraining measures. The perception of informal coercion often refers to a bundle of measures, the experience of which is shaped by the interaction between patient and healthcare providers. We wish to include the positive narratives in our study as a feedback for the team about quality of care and professionalism, but also regarding the relationship as described in the study on “thank you letters” by Herbland et al. [56]. Even if a wide range of mental, physical, social, and functional sequelae remain after ICU discharge, summarised as post intensive care syndrome (PICS), many ICU survivors may experience positive emotions and fulfilment [57]. Still, most patients stated that they did not wish to experience an ICU setting again.

In a next step, an experience-based co-design (EBCD) [58] would be helpful for the health care teams to better understand patients' perceptions of possible coercion in the ICU and then to develop counter-strategies.

### Limitations

Our study has limitations. First, we describe and discuss memories of patients that have been influenced by circumstances, illness and subsequent information. Additionally, the study is defined by the willingness of patients and relatives to tell their story. Unfortunately, no relatives of deceased patients could be identified who were willing to share their memories to balance out the stories of survivors and their relatives.

## Conclusions

Formal coercion in the form of restraining measures, restricted freedom of movement and coercive treatment in the ICU exists and is perceived as such by patients. In order to capture the full range of patient experiences, it is necessary to broaden the concept of coercion to include less obvious forms of informal coercion that may arise in situations of dependency. The loss of autonomy and dignity can enhance the sense of informal coercion perceived by a patient.

Beyond semantics, the identification of what we have termed "informal coercion" from the patient's perspective is intended to encourage a sensitive perception and to foster a critical and caring approach to possible forms of coercion and to put forth ways to improve patient care in the ICU.

An ethical climate characterised by respect for the patient, relatives and the healthcare team can be helpful in reducing coercive measures and the moral distress for those applying them. Treatment measures must be seen not only in terms of their potential to reduce mortality, but also with a view to how they shape the experience and memories of patients. Negative recollections may persist long after discharge and negatively affect the patient’s quality of life (post intensive care syndrome). Integrating patients’ advice such as "don't touch me", “aggression comes from fear” or “I was very happy when they stopped the morphine”, “they should approach people slowly” can make an important contribution to the ethical management of patients in the ICU.

## Supplementary Information

**Additional file 1.**. The topic guide included three parts.

## Data Availability

The datasets used and/or analysed during the current study are available from the corresponding author on reasonable request.
