# Supplementary material for: What does coercion in intensive care mean for patients and their relatives? A thematic qualitative study
Source: BMC Med Ethics. 2022 Feb 5;23:9. doi: 10.1186/s12910-022-00748-1 (PMC8817558; doi:10.1186/s12910-022-00748-1)
Supplement: Supplementary file 1 — Additional file 1.. The topic guide included three parts. [file 12910_2022_748_MOESM1_ESM.docx]

Appendix: interview guide


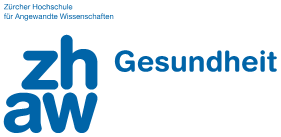

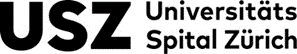

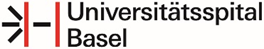

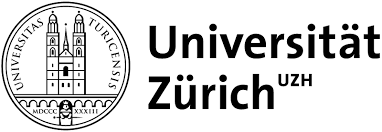


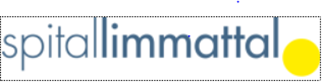

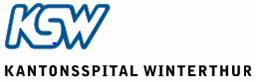


**Interview guide for patients and relatives after ICU discharge**

**Date:**

**Informed Consent:**

**Name:**

**Age:**

**Gender:**

**ICU length of stay**

**Participants:**

**Interviewer:**

Time: ca. 60 min

**Introduction:**

- Introduce yourself and establish confidence and an open and empathic communication, avoid judgement behaviour,
- Gain Informed Consent
- Describe the timing and procedure of the interview. Establish communication following the guideline. Invite the participant to speak freely and do not force answers. The participant must be allowed to avoid sensitive topics or skip specific questions.

Establish: video-/audio record

**Interview**

First, I’d like to thank you very much for joining this study. We know that experiences made in the ICU can be very delicate and personal - so we can take a break or stop recording at any time.

Today I’d like to ask you some questions on different topics relating to your stay in the ICU. Please do take your time considering your answer.

**Interview:** open and unforced talk:

Can you please tell us if your intervention was part of an elective treatment or performed under emergency circumstances?

**Relatives:** Remembering the time in the intensive care unit, can you tell us your experiences?

**Patients:** Remembering the time in the intensive care unit, can you tell us your experiences? What are your memories?

| Patient | Relative |
| --- | --- |
| Do you remember situations in which you felt helpless?   - Could you describe these situations? - Emotionally, how would you describe those situations if referring to a friend/relative? | Do you remember situations in which you felt helpless?   - Could you describe these situations? - Emotionally, how would you describe those situations if referring to a friend/relative? |
| Do you remember situations when you were **unable to move** yourself?   - How do you remember this situation? What kind of emotions do you link to those experiences? |  |
| Did you have the feeling that you would have **decided against** some of the treatment approaches, that something was going on you **did not want**? (esp. moving, rehabilitation measures)   - Is there a specific situation that comes to mind associated with what we’ve just been talking about? Situations with restraint? | Did you have the feeling your relative would have **decided against** the treatment approaches, that something was going on your relative **did not want**? (esp. moving, rehabilitation measures)   - Which situation do you remember and how were your emotions? |
| Could you please describe a situation in which you felt **dehumanised or horrible/terrible** as a result? Is there anything specific you link to those situations?   - Do you recall bad dreams, or having nightmares, pain or being near death? - Describe your individual emotions in these situations. - Emotions like being a tool? | Could you please describe a situation in which you felt bad?   - What do you link with these unhappy/bad feelings? - Could you please describe your individual emotions in these situations? - How was your experience – that of being an unheard relative? |
| Do you remember situations of well-being?   - What do you link with these emotions? | Do you remember situations of well-being?   - What do you link with these emotions? |
| Has there been a situation in which you felt desperate and lonely? | Has there been a situation in which you felt desperate and lonely? |
| Do you recall a shared decision-making process?   - Could you take part? | How do you recall the shared decision-making process?   - Could you take part? |
| When thinking back on your time in the ICU, how do you feel?   - Would you like to offer some advice and/or feedback to the nurses, physicians and team on the ward? | When thinking back on this time when your relative was in the ICU, how do you feel?   - Would you like to offer some advice and/or feedback to the nurses, physicians and team on the ward? |
